# Correction: Concurrent coverage and determinants of vitamin A supplementation and deworming among children aged 12–59 months in 15 Sub-Saharan African countries

**DOI:** 10.1371/journal.pone.0344650

**Published:** 2026-03-09

**Authors:** Thomas Kidanemariam Yewodiaw, Mequanent Dessie Bitewa, Abiyu Abadi Tareke

The second author’s name is spelled incorrectly. The correct name is: Mequanent Dessie Bitewa.
