# Supplementary material for: Concurrent coverage and determinants of vitamin A supplementation and deworming among children aged 12–59 months in 15 Sub-Saharan African countries
Source: PLoS One. 2026 Jan 15;21(1):e0339759. doi: 10.1371/journal.pone.0339759 (PMC12806840; doi:10.1371/journal.pone.0339759)
Supplement: Attachment — Submitted filename: Response to Reviewers.pdf [file pone.0339759.s004.pdf]

Response to Reviewers' Comments

We thank the reviewers for their thoughtful comments, which helped improve the clarity, quality, and programmatic relevance of our manuscript. Below, we provide a detailed point-by-point response.

Abstract

Comment 1: Format the text; specify software and significance/confidence level.
Response:

- The abstract has been reformatted for consistent font and style.
- The methods section now specifies: **Stata version 17.0** was used, with **95% confidence intervals (CI)** and **$p<0.05$** considered statistically significant.

Introduction

Comment 2: Avoid inconsistent geographic references (Sub-Saharan Africa vs East Africa). Example line 60.
Response:

- All geographic references have been standardized to **Sub-Saharan Africa** unless specifically indicated.
- The sentence on *Ascaris lumbricoides* now reads:
  "In Sub-Saharan Africa, soil-transmitted helminths such as Ascaris lumbricoides affect approximately 28–30% of children, contributing to anemia and growth retardation."

Methods

Comment 3: Why include only children ≥12 months?
Response:

- Children aged **12–59 months** were included because DHS reliably collects VAS/DW data for this age group. This reduces recall bias for younger children (6–11 months).

Comment 4: Sample size discrepancy (10,725 vs 107,725).
Response:

- Corrected throughout the manuscript: the total sample is **107,725 children**.

Comment 5: Address maternal recall bias.
Response:

- Added that maternal recall may introduce bias; **child health cards were used for verification when available**.

Comment 6: Clarify community variable definitions.
Response:

- Community-level variables (e.g., education, poverty) were **aggregated at the cluster level** and dichotomized using the **national median**.

Comment 7: Criteria for variable selection in a multilevel model.
Response:

- Variables with **p<0.20 in bivariate analysis** and theoretically relevant variables were included in the multilevel model.

Results

Comment 8: Reduce redundancy between text and tables.
Response:

- Results section revised to **summarize key points in text** and emphasize tables/figures for details.

Comment 9: Improve Figure 1.
Response:

- Figure 1 enhanced with **standardized color coding and clear country labels** for better comparison.

Comment 10: Include measures of heterogeneity (ICC/MOR).
Response:

- **ICC and MOR** are now reported in the Results to quantify country-level variation.

Comment 11: Typographical errors.
Response:

- Corrected "13% deference" to **"13% difference."**

Discussion

Comment 12: Reduce repetition; focus on programmatic implications.
Response:

- Discussion condensed, emphasizing **policy relevance, integration with immunization, ANC, and child health campaigns**.

Comment 13: Urban/rural differences.
Response:

- Urban areas showed lower co-coverage than expected; discussed factors include **informal settlements, limited community outreach, and system access issues**.

Comment 14: Include programmatic limitations.
Response:

- Added a limitation: **data on stock-outs, campaign timing, and delivery platforms were not available**, potentially affecting coverage estimates.

Comment 15: Include table/narrative on VAS/DW delivery platforms.
Response:

- Added a new table summarizing delivery platforms by country:
  - **Tanzania:** Biannual Child Health Days only
  - **Senegal:** Routine VAS only
  - **Mozambique/Kenya:** Routine health system + CHW outreach

Comment 16: Reduce repetitive references to Mozambique.
Response:

- Mozambique discussion now integrated into **Moderate co-coverage category**, highlighting operational gaps. Redundant statements removed.

Comment 17: Differentiate recommendations for low, moderate, high coverage countries.
Response:

- Recommendations now **tailored to coverage categories**, considering delivery platforms and programmatic gaps.

Comment 18: Supply/stock-out limitations.
Response:

- Added note: **Vitamin A capsules largely secured via global donation programs, while deworming tablets are often scarce**, which may influence coverage.

Conclusion

Comment 19: Strengthen the link between categorization and recommendations.
Response:

- Conclusion revised to emphasize that **categorizing countries by co-coverage allows targeted interventions**, with recommendations specific to low, moderate, and high coverage contexts based on delivery platform and system performance.

We appreciate the reviewers' suggestions and believe the manuscript is now stronger in clarity, scientific rigor, and programmatic relevance.
