# Supplementary material for: Concurrent coverage and determinants of vitamin A supplementation and deworming among children aged 12–59 months in 15 Sub-Saharan African countries
Source: PLoS One. 2026 Jan 15;21(1):e0339759. doi: 10.1371/journal.pone.0339759 (PMC12806840; doi:10.1371/journal.pone.0339759)
Supplement: Attachment — Submitted filename: Response to Reviewers and Editor up.pdf [file pone.0339759.s006.pdf]

Response to Reviewers and Editor

Manuscript ID: PONE-D-25-47082R2
Title: Concurrent Coverage and Determinants of Vitamin A Supplementation and Deworming Among Children Aged 12–59 Months in 15 Sub-Saharan African Countries
Authors: Thomas Kidanemariam Yewodiaw et al.

On behalf of all authors

General Response

We sincerely thank the editor and reviewers for their constructive comments. We have carefully revised the manuscript to address all points raised. Below, we provide a detailed, point-by-point response indicating the changes made, including specific line numbers and figure/table references.

Editor Comments and Responses

Editor Comment	Response / Action Taken	Location / Notes
**1. Table 2 references:** Many references in Table 2 (with URLs); should they appear in the bibliography?	All references cited in Table 2 have been added to the main bibliography following PLOS ONE style. URLs included; formatting checked.	Table 2, References section
**2. Citation style:** round brackets `()` used; PLOS prefers square brackets `[]`.	All in-text citations updated to square brackets `[]` per PLOS ONE style.	Lines throughout the manuscript; bibliography cross-checked
**3. Acronyms inconsistent:** Examples include Sub-Saharan African vs Sub-Saharan Africa; VAD, VAS, DW. Lines: 55, 62, 65, 80, 86, 92, 94, 98, 144, 188, 228, 257, 262, 267, 275, 283, 381, 382, 390.	- Sub-Saharan Africa (SSA) was used consistently; "Sub-Saharan African" was corrected were inappropriate.<br>- VAD is defined at first mention (line 55).<br>- VAS (Vitamin A Supplementation) and DW (Deworming) acronyms are now used consistently throughout the manuscript and figures.<br>- Kids Recode (KR) acronym removed where rarely used.	Lines as above; Figures 1–3

**4. Other grammatical issues:** Line 141 missing; line 170 "determinant's" → "determinants"; clarify "among what?"	- Line 141 corrected with a closing parenthesis.<br>- Line 170 corrected to "determinants of Vitamin A Supplementation and Deworming among children aged 12–59 months".<br>- Minor grammar, punctuation, and sentence structure errors corrected throughout.	Lines 141, 170; Methods, Results sections
**5. Variable codes in Methods (h34, h43):** Too detailed?	DHS variable codes replaced with descriptive names for clarity while maintaining reproducibility. Reference to DHS survey files retained.	Methods section, lines 130–150
**6. Capitalization in subheadings and titles:** Ensure consistency; likely first word + proper nouns.	All subheadings, figure captions, and table titles standardized to PLOS ONE style (first word + proper nouns).	Subheadings throughout manuscript; Figures 1–3; Tables 1–3
**7. Vitamin A terminology:** Multiple variants (Figure 1 title, lines 170, 197, 260).	Standardized all instances to "Vitamin A Supplementation (VAS)" for supplementation, "Vitamin A Deficiency (VAD)" for deficiency.	Lines 55, 170, 197, 260; Figures 1–3

Figures and Tables

- Figures checked using **NAAS tool** for compliance with PLOS ONE requirements: [PLOS ONE NAAS Tool]
- Figure captions standardized for capitalization, clarity, and consistent acronyms.
- All tables formatted to PLOS ONE style, including reference inclusion in Table 2.

Additional Notes

1. Protocols and methods clarified for reproducibility; unnecessary DHS variable codes removed.
2. Abbreviations are defined at first use and applied consistently throughout text and figures.
3. Minor typos, punctuation, and grammar corrected throughout the manuscript.

We thank the editor and reviewers again for their detailed and constructive feedback. We believe the revised manuscript addresses all points raised and is now suitable for publication in PLOS ONE
