# Supplementary material for: Concurrent coverage and determinants of vitamin A supplementation and deworming among children aged 12–59 months in 15 Sub-Saharan African countries
Source: PLoS One. 2026 Jan 15;21(1):e0339759. doi: 10.1371/journal.pone.0339759 (PMC12806840; doi:10.1371/journal.pone.0339759)
Supplement: Attachment — Submitted filename: Response to Reviewers-updated.pdf [file pone.0339759.s008.pdf]

Response to Reviewers
Manuscript ID: PONE-D-25-47082R4
Title: *Concurrent Coverage and Determinants of Vitamin A Supplementation and Deworming Among Children Aged 12–59 Months in 15 Sub-Saharan African Countries*
Author: Thomas Kidanemariam Yewodiaw

Dear Dr. Horton and Reviewers,

We thank you for the constructive feedback on our manuscript. We have carefully revised the manuscript to address all points raised. Below, we provide a point-by-point response. Changes in the manuscript are highlighted in the revised version.

Editor Comments and Our Responses

Comment 1: "There are still issues with abbreviations (see below). I suggest that in the text, you should define an acronym once and ONCE ONLY, and thereafter use the acronym. Do not define it again in section/figure/table titles."

Response:
We have carefully reviewed the manuscript and corrected all issues with abbreviations. All acronyms are now defined once at their first occurrence in the main text. Full terms are used in section titles, table titles, and figure titles without repeating the acronym.

- Examples of corrections:
  - Line 60: "soil-transmitted helminths (STH)" defined once in text; "Soil-Transmitted Helminths" used in section title without repeating acronym.
  - Lines 63, 65, 81, 82, 87, 90, 91, 180, 181, 210, 213, 229, 264, 265, 304, 305, 313, 354, 355, 391: all section/figure/table titles now use full terms only.

Comment 2: "Although some of the issues in the references have been fixed, please note that PLOS ONE uses the ICMJE referencing style, using abbreviated journal names and appropriate capitalization of journal titles."

Response:
We have reviewed all references and corrected them according to the ICMJE style, including:

- Abbreviating journal names using NLM recommendations.
- Correcting capitalization of journal titles.
- Verifying that all references are accurate and up-to-date.

For example:

- Reference 13: Corrected to “BMJ” with proper capitalization.
- References 1, 2, and others updated to match ICJME style.
- DOI numbers added where available.

Additional Changes Made:

1. Minor grammatical and formatting corrections throughout the manuscript.
2. Ensured consistency in reporting vitamin A supplementation (VAS) and deworming coverage.
3. Updated figure captions for clarity and compliance with PLOS ONE guidelines.

We believe that these revisions have addressed all the points raised and have improved the clarity and quality of the manuscript. We sincerely thank the editor and reviewers for their constructive comments and suggestions.

Sincerely,
Thomas Kidanemariam Yewodiaw
Gondar, Ethiopia
