# Supplementary material for: Concurrent coverage and determinants of vitamin A supplementation and deworming among children aged 12–59 months in 15 Sub-Saharan African countries
Source: PLoS One. 2026 Jan 15;21(1):e0339759. doi: 10.1371/journal.pone.0339759 (PMC12806840; doi:10.1371/journal.pone.0339759)
Supplement: Attachment — Submitted filename: Review - PLOS ONE (1).docx [file pone.0339759.s001.docx]

**Manuscript ID:** PONE-D-25-47082

**Title:** Concurrent Coverage and Determinants of Vitamin A Supplementation and Deworming Among Children Aged 12–59 Months in 15 Sub-Saharan African Countries

**Abstract**

- Format the text, as the font is not uniform.
- Inform in the methods the software used to analyse the data, including its version and the significance level or confidence interval used.

**Introduction**

- It's important to avoid back-and-forth geographic delimitations throughout the text. In some passages, Sub-Saharan Africa is mentioned, then East Africa, before returning to Sub-Saharan Africa. This can cause confusion for the reader. An example is in line 60, in the sentence about Ascaris lumbricoides, which affects approximately 28% to 30% of children: it's unclear whether this data refers specifically to Sub-Saharan Africa or East Africa.

**Methods**

- Why did the study only include children 12 months and older, if the recommendation is that vitamin A supplementation begin at 6 months of age?
- Note: The abstract and results list 107,725 children, but the methods (line 86 of the PDF) state "10,725" – this should be revised for consistency.
- Explain how the issue of recall bias in maternal information was addressed (a clear limitation).
- Clarify how community variables were defined (e.g., categorization by national median).
- Reinforce criteria for variable selection in the multilevel model: was p<0.20 adopted in the bivariate analyses, or were all variables included based on theoretical grounds?

**Results**

- The presentation of the results is clear, but there is redundancy between the text and tables. I suggest summarizing the text more and emphasizing tables/figures.
- Figures: Figure 1 could be improved graphically to facilitate comparative reading between countries.
- Include measures of heterogeneity between countries (ICC or MOR) in the summary as well, as this is an important finding.
- Review minor typographical errors: "13% deference" (line 236) should be "difference."

**Discussion**

- The discussion is comprehensive, but lengthy in some sections and repetitive compared to the introduction. It is recommended to summarize and focus on programmatic implications.
- Emphasize the urban/rural differences found, as the result contrasts with what was expected (lower coverage in urban areas).
- Highlight better policy implications, integration with immunization, ANC, and child health campaigns.
- Limitations: In addition to the cross-sectional design and recall bias, it is worth noting that programmatic variables (e.g., availability of inputs, campaign schedule) were not available.
