# Supplementary material for: Concurrent coverage and determinants of vitamin A supplementation and deworming among children aged 12–59 months in 15 Sub-Saharan African countries
Source: PLoS One. 2026 Jan 15;21(1):e0339759. doi: 10.1371/journal.pone.0339759 (PMC12806840; doi:10.1371/journal.pone.0339759)
Supplement: Attachment — Submitted filename: PLOS review_Oct2025 (1).docx [file pone.0339759.s002.docx]

1) Overall, really appreciate the analysis of co-coverage and the overall messaging of the paper. It is very relevant and offers nice insight into the needs for health system strengthening to ensure equitable access and coverage for all children with all services, not just some services.
2) The paper would benefit from a table or narrative describing the VAS/DW delivery platform used at the time of the DHS coverage data reported. For example, Tanzania delivers VAS via biannual Child Health Days only, no routine VAS; Senegal is routine VAS only – no campaigns. An example is that even within a “category” there is diversity - In the Moderate category Mozambique and Kenya deliver VAS via routine health systems with active community health worker outreach, whereas Tanzania delivers VAS and DW via biannual Child Health Days. This will improve/contextualize the analysis of causes, and recommendations for improvement. The general statement in all three categories of low, moderate, high coverage countries that improved campaign alignment for example is not helpful as most of the countries used in the analysis do not all deliver the interventions via campaigns (unless they did the year in which the DHS data was collected – this needs validating).
3) Calling out Mozambique again in lines 297-299 “Mozambique’s moderate co-coverage highlights room for improvement, with operational gaps in synchronized campaigns, monitoring, and community outreach (35, 38).” Then again in Line 301 “Strengthening Mozambique’s co-coverage will require adopting best practices such as coordinated campaigns, robust supply chains, and active community engagement(40). “ is out of place and is repetitive to what is earlier stated in the Lines 285-286 for Moderate co-covg countries. Suggest if authors wish to use Mozambique to go into greater detail, that this be all part of the Moderate category discussion.
4) This line: 299-300 “Low co-coverage countries face systemic barriers, while high performers offer models of effective integration.” seems to be out of place/repeats what is above in Lines 293-295.
5) This conclusion stated in line 303 “Categorizing countries by co-coverage allows targeted strategies to optimize child health outcomes across Sub-Saharan Africa.” Is a valuable conclusion, however is not strongly supported by the narrative presented. The low, moderate and high seem to have very similar causes and same recommendations, pasted here are the lines I am referring to:

- 292-293: Low: Systematic planning, aligned campaigns, and strengthened health systems are essential to improve co-coverage
- 285-286: Moderate: synchronized delivery, stronger community engagement, and better resource allocation have been shown to improve co-coverage in such contexts
- 275-276 High co-cvg: well-planned, integrated campaigns enhance simultaneous uptake, maximize health benefits, and improve cost-effectiveness

Recommend that the reasons for low, moderate, and high co coverage go a little bit deeper (perhaps by looking at the delivery platforms in concert with the coverage data), and try to make the recommendations a bit more specific to the low, mod, high contexts – this will strengthen the conclusion.

6) Lastly, suggest the authors mention the limitation of line of sight on supply/stock outs when looking at these data. The impact of Deworming medication stock outs on coverage all across SSA cannot be underestimated. Vitamin A capsules are largely secured in all countries through the global capsule donation program led by Nutrition International in partnership with UNICEF, however deworming tablets are scarce.
