# Supplementary material for: Concurrent coverage and determinants of vitamin A supplementation and deworming among children aged 12–59 months in 15 Sub-Saharan African countries
Source: PLoS One. 2026 Jan 15;21(1):e0339759. doi: 10.1371/journal.pone.0339759 (PMC12806840; doi:10.1371/journal.pone.0339759)
Supplement: Attachment — Submitted filename: Response to Editor and Reviewers.docx [file pone.0339759.s005.docx]

**Response to Editor and Reviewers**

**Manuscript ID:** PONE-D-25-47082R1
**Title:** ***Concurrent Coverage and Determinants of Vitamin A Supplementation and Deworming Among Children Aged 12–59 Months in 15 Sub-Saharan African Countries***

Dear Dr. Susan Horton and the Editorial Team,

We sincerely thank you for your careful review and constructive comments on our manuscript. We appreciate your valuable suggestions that have greatly improved the clarity, grammar, and overall quality of the paper. Below, we provide a detailed, point-by-point response to each of the editorial comments and indicate the corresponding changes made in the revised version. Line numbers refer to the *Revised Manuscript with Track Changes* version.

**1. Table 2: “Where is this?”**

**Response:**
We apologize for the omission. Table 2 has now been included in the revised manuscript and properly referenced in the text (Lines 162–165).

**2. Use of abbreviations – “Sub-Saharan Africa (SSA)”**

**Comment:** Use “Sub-Saharan Africa (SSA)” instead of “Sub-Saharan Africa Countries (SSA)” and apply consistently throughout the manuscript.
**Response:**
We have replaced all instances of “Sub-Saharan Africa Countries (SSA)” with “Sub-Saharan Africa (SSA)” and ensured consistent usage throughout the manuscript (Lines 61, 67, 69, 71, 93, 175, and 200) on old manuscript.
Where appropriate, “15 sub-Saharan African countries” is used instead of “SSA countries.”

**3. Repetition of “Demographic and Health Surveys (DHS)”**

**Comment:** Avoid repeatedly spelling out the full name once the abbreviation is introduced.
**Response:**
We now spell out “Demographic and Health Surveys (DHS)” only once at its first appearance (Line 18). All subsequent references use “DHS” only (e.g., Lines 57 and elsewhere).

**4. Remove “Abbreviations” list at the end of the text**

**Response:**
As per PLOS ONE style, the “Abbreviations” section at the end has been deleted.

**5. Reference formatting – add space before reference numbers**

**Response:**
We have formatted all in-text citations according to PLOS style, ensuring a space precedes each reference number (e.g., “…in SSA [1]”).

**6. Capitalization consistency in section headings and table titles**

**Response:**
We standardized capitalization across all section headings, table titles, and figure captions using Title Case (e.g., “Determinants of Co-Coverage of Vitamin A and Deworming in Sub-Saharan Africa”).

**7. Grammar and style corrections**

**Response:**
We have made all the grammatical and stylistic corrections suggested by the editor. Specific changes include:

| **Line (Old)** | **Original Text** | **Revised Text** |
| --- | --- | --- |
| 32 | “receiving” | “received” |
| 36–37 | “Vitamin A supplementation alone low coverage in Gabon (15.7%) and Sierra Leone (16.5%) to high coverage in Rwanda (89.1%)” | “Vitamin A supplementation coverage was lowest in Gabon (15.7%) and Sierra Leone (16.5%) and highest in Rwanda (89.1%).” |
| 52 | “exceeding” | “leading” |
| 165 | “Co-coverage” | “Co-coverage of” |
| 167 | “Determinant’s of Vit A and Deworming Co-Coverage among in Sub-Saharan Africa” | “Determinants of vitamin A and deworming co-coverage in Sub-Saharan Africa.” |
| 182 | “of” | “in” |
| 192–198 | Repetitive text | Deleted |
| 199 | “deworming” | “coverage of vitamin A and deworming individually” |
| 322 | “be achieve” | “achieve” |
| 395 | Bold “of” | Bold removed |

All other minor grammatical inconsistencies have been corrected throughout the manuscript.

**8. General professional editing**

**Response:**
We thoroughly proofread and edited the entire manuscript for grammar, punctuation, and flow. We also ran a grammar check using Microsoft Word Editor and Grammarly to ensure professional writing quality and adherence to *PLOS ONE* standards.

**9. Figures and Tables**

**Response:**
All tables and figures were checked for consistency in titles, captions, and numbering. Figures comply with *PLOS ONE* technical and formatting guidelines.

**10. References**

**Response:**
We reviewed all references for completeness and accuracy. None of the cited articles have been retracted. Minor formatting corrections were made according to *PLOS ONE* reference style.
